# Biogenic Synthesis and Characterization of Chitosan-CuO Nanocomposite and Evaluation of Antibacterial Activity against Gram-Positive and -Negative Bacteria

**DOI:** 10.3390/polym14091832

**Published:** 2022-04-29

**Authors:** Peace Saviour Umoren, Doga Kavaz, Alexis Nzila, Saravanan Sankaran Sankaran, Saviour A. Umoren

**Affiliations:** 1 Department of Bioengineering, Cyprus International University, via Mersin 10, Nicosia 98258, Turkey; princespeace@yahoo.com; 2 Department of Bioengineering, King Fahd University of Petroleum and Minerals (KFUPM), Dhahran 31261, Saudi Arabia; alexisnzila@kfupm.edu.sa (A.N.); saravanan@kfupm.edu.sa (S.S.S.); 3 Interdisciplinary Research Center for Membranes and Water Security, King Fahd University of Petroleum and Minerals (KFUPM), Dhahran 31261, Saudi Arabia; 4 Interdisciplinary Research Center for Advanced Materials, King Fahd University of Petroleum and Minerals (KFUPM), Dhahran 31261, Saudi Arabia

**Keywords:** chitosan, copper oxide, olive leaf extract, nanocomposite, antibacterial activity

## Abstract

Chitosan-copper oxide (CHT-CuO) nanocomposite was synthesized using olive leaf extract (OLE) as reducing agent and CuSO_4_⋅5H_2_O as precursor. CHT-CuO nanocomposite was prepared using an in situ method in which OLE was added to a solution of chitosan and CuSO_4_⋅5H_2_O mixture in the ratio of 1:5 (*v*/*v*) and heated at a temperature of 90 °C. The obtained CHT-CuO nanocomposite was characterized using field emission scanning electron microscopy (FE-SEM), X-ray diffraction (XRD), ultraviolet-visible (UV-Vis) spectrophotometry, energy-dispersive X-ray spectroscopy (EDAX), Fourier transform infrared spectroscopy (FTIR), and high-resolution transmission electron microscopy (TEM). TEM results indicated that CHT-CuO nanocomposite are spherical in shape with size ranging from 3.5 to 6.0 nm. Antibacterial activity of the synthesized nanocomposites was evaluated against Gram-positive (*Bacillus cereus*, *Staphyloccous haemolytica* and *Micrococcus Luteus*) and Gram-negative (*Escherichia coli*, *Pseudomonas citronellolis*, *Pseudomonas aeruginosa*, *kliebisella* sp., *Bradyrhizobium japonicum* and *Ralstonia pickettii*) species by cup platting or disc diffusion method. Overall, against all tested bacterial strains, the diameters of the inhibition zone of the three nanocomposites fell between 6 and 24 mm, and the order of the antimicrobial activity was as follows: CuO-1.0 > CuO-0.5 > CuO-2.0. The reference antibiotic amoxicillin and ciprofloxacin showed greater activity based on the diameter of zones of inhibition (between 15–32 mm) except for *S. heamolytica* and *P. citronellolis* bacteria strains. The nanocomposites MIC/MBC were between 0.1 and 0.01% against all tested bacteria, except *S. heamolityca* (>0.1%). Based on MIC/MBC values, CuO-0.5 and CuO-1.0 were more active than CuO-2.0, in line with the observations from the disc diffusion experiment. The findings indicate that these nanocomposites are efficacious against bacteria; however, Gram-positive bacteria were less susceptible. The synthesized CHT-CuO nanocomposite shows promising antimicrobial activities and could be utilized as an antibacterial agent in packaging and medical applications.

## 1. Introduction

Micro-organisms are important in a wide range of life-sustaining functions. On the other hand, some are pathogenic, meaning they can cause illness and even death. Despite the availability of numerous antibiotics for the treatment of bacterial ailments, new infectious illnesses and bacterial resistance continue to emerge [1]. Antibiotic resistance is rising at an unprecedented rate, necessitating the development of novel antimicrobials [2]. Furthermore, recent interest in identifying safe and natural antibiotic substitutes [3,4] has been fuelled by environmental concerns about some antibiotics [1]. Antibiotic misuse has resulted in bacteria developing a biofilm-forming defensive mechanism [3,4], making medications less effective. As a result, several researchers are concentrating on developing further effective antimicrobial drugs to manage multidrug-resistant diseases in order to reduce bacterial growth [2,4].

Nanotechnology has given rise to a slew of novel antibacterial solutions. The nanoparticles’ modest size makes them ideal for carrying out antibacterial biological operations [5]. Metal nanoparticles, such as silver, zinc, copper, and iron, have shown great promise as bactericidal and fungicidal components, proving their potential as effective antibiotic therapies in wound care and other medical conditions [6]. Nanomaterials and nanocomposites are a promising platform for alternative strategies to manage bacterial ailments, as they provide long-term antibacterial activity with minimal toxicity, as opposed to tiny-molecule antimicrobial drugs, which have short-term activity and are hazardous to the environment. The antimicrobial nanoparticle physically damages the organism’s cell membranes, preventing the formation of drug-resistant bacteria [7]. Cu and CuO nanoparticles (CuO NPs) have long been used in biological systems to prevent harmful bacteria and algae from growing [8,9,10]. 

For the synthesis of nanoparticles, diverse approaches have been used, including chemical, physical, and biological synthesis [11]. Nanoparticles are produced by reducing metallic compounds with any biological or microbe, plant, or their extracts [12]. Because it is eco-friendly, economical, simple, and suitable for large-scale manufacturing, biological preparation of metal nanoparticles utilizing plant extracts is recommended [13,14]. Plant extracts are utilized to make metal nanoparticles as an alternative to chemical, physical, and microbiological methods [13]. Metal nanoparticles made with plant extracts have been demonstrated to be very durable and harmless for packing and human medicinal uses [14,15,16], in addition to the benefits stated above. Furthermore, biologically generated nanoparticles do not require any additional stabilizing agents because the plants used act as capping and stabilizing agents [17]. Biological nanoparticle preparation is a bottom-up strategy in which reductants and stabilizers aid in the creation of nanoparticles. Plant phytochemicals or microbial enzymes that serve as reductants are typically responsible for the reduction of metal compounds [18]. Plant extracts containing bioactive alkaloids, phenolic acids, polyphenols, proteins, and carbohydrates are critical first in the reduction and then in the stabilization of metallic ions [19,20].

Nanocomposites are a type of hybrid material that includes nanoscale reinforcements and a matrix. Nanoscale elements are made in a variety of forms and geometries, with typical dimensions of less than 100 nm. Nanoparticles and nanofibers are two nanoscale constituents commonly employed in nanocomposite systems as reinforcement. There are three types of nanocomposite materials: polymeric matrix nanocomposites (PMNCs), metallic matrix nanocomposites (MMNCs), and ceramic matrix nanocomposites (CMNCs). PMNCs and MMNCs have been extensively researched over the last two decades because of their superior mechanical, electrical, thermal, and chemical characteristics [21,22,23]. When particles are diminished in size from a micrometre to a nanometre, their characteristics might drastically change. Electrical conductivity, rigidity, active surface area, chemical reactivity, and biological activity, for example, have all been acknowledged to change. The bactericidal activity of metal and metal oxide nanoparticles and nanocomposites has been linked to their size and giant surface-area-to-volume ratio [24,25,26].

Chitosan is a linear polysaccharide made up of randomly scattered N-acetyl-D-glucosamine and linked D-glucosamine. It is made by treating the chitin shells of shrimp and other crustaceans with an alkaline material, such as sodium hydroxide (NaOH). Due to its bioactive features, such as biodegradability, non-toxicity, biocompatibility, haemostatic action, drug transport, and antibacterial properties, chitosan has a variety of commercial, pharmaceutical, biological, and food applications [27,28,29,30,31]. The availability of protonated groups in the polymer backbone, as well as ionic interactions between the charged groups and bacteria wall components, contribute to its antibacterial activity. Protonation of the –NH_2_ functional groups in acid solution or structural changes (e.g., methylation, sulfonation, etc.) can produce charges on the chitosan backbone [27,30]. As a result, the peptidoglycans in the microbe wall are hydrolysed, causing intracellular electrolyte leakage and the micro-organism’s death [27].

Polymer–metal/metal oxide nanocomposites are new type of hybrid material that have the potential to increase functional qualities dramatically (e.g., biological, electrical conductivity) and enhance antimicrobial properties [32,33]. Metal oxide nanoparticles coupled with chitosan have been demonstrated to be an outstanding biocompatible substance in recent investigations [34,35]. Chitosan–NiO (CS-NiO) and chitosan–MgO (CS-MgO) have been shown to have antibacterial activity against *E. coli* and *S. aureus* bacteria strains [36]. The findings revealed that all of the samples had antibacterial properties against the tested bacteria strains. After 12 h of incubation, the CS-NiO showed greater efficacy as an antibacterial agent, reducing *S. aureus* and *E. coli* viabilities to 2–8%. According to the findings, CS NiO nanocomposites have the potential to be employed as an effective antibacterial agent against dangerous bacterial infections. An environmentally friendly biological synthesis of chitosan/copper oxide (CS-CuO) a nanocomposite utilizing rutin and its antiproliferative efficacy against human lung cancer cell line A549 can be found in the literature [37]. The prepared CS-CuO nanocomposite was reported to have concentration-dependent antiproliferative action against A549 cancer cells, with an IC50 value of 20 0.50 g/mL. Furthermore, using the AO/EtBr fluorescence labelling approach, the produced nanocomposite promotes apoptosis in treated A549 cancer cells. Bharathi et al. [38] also described the simple synthesis of a chitosan-FeO nanocomposite with antibacterial action against Gram-positive and Gram-negative bacterial pathogens.

CuO NPs produced from diverse plant-based materials (extracts) have been shown to have antimicrobial activity in the literature [39,40,41,42,43,44]. There is no published report on the antibacterial activity of chitosan-CuO nanocomposites produced using olive leaf extract as reducing agents against Gram-negative and Gram-positive bacteria. We adopted a facile technique to synthesize chitosan-CuO nanocomposites utilizing aqueous olive leaf extract as a reducing agent. The advantage is that plant-extract-mediated synthesis is the fastest, cheapest, and most sustainable of the numerous green synthesis processes. Furthermore, using antimicrobial plant extract as an in situ reducing and capping agent/group aids in the synthesis of nanoparticles with enhanced antimicrobial activity.

## 2. Materials and Methods

### 2.1. Materials

Chitosan (Merck, Kenilworth, NJ, USA) (MW: 50,000–190,000 Da, degree of deacetylation: 75–85 percent), CuSO_4_⋅5H_2_O (Merck), and CH_3_COOH (Merck) were utilized as received without additional purification. Fresh olive leaves (OLE) were picked near the Haspolat campus of Cyprus International University (CIU). *Bacillus licheniformis* (KF609498), *Staphylococcus haemolytic* (MN388897), *Bacillus cereus* (MN888756), and *Micrococcus luteus* (MN888755) were the Gram-positive bacteria strains used, whereas the Gram-negative bacteria strains were *Pseudomonas aeruginosa* (gene accession number GI482716237), *Pseudomonas citronellolis* (ATCC 25992), *Bacillus japonicum*, *Ralstonia pickettii*, and *Klebisella* sp. (reference on ATCC global resource). The National Center of Biotechnology Institute has these gene accession numbers (NCBI) [45,46]. 

Different methods were used to prepare the samples for different investigations. For instance, in XRD analysis, the nanoparticles were separated. For TEM, FTIR, and FE-SEM analyses, a simple drying procedure was applied to obtain the solid residue sample used for the analysis, whereas the colloidal solution of the nanocomposite was directly used for UV-vis and Zeta potential analyses. 

### 2.2. Extraction of Plant Leaves

Olive leaves were properly cleaned, then sun-dried for 14 days before being pulverized into powder. To prepare the aqueous olive leaf extract, 5.0 g of the powdered leaf was introduced into 250 mL of distilled H_2_O and heated to 100 °C under constant stirring at 200 rpm for 3 h. It was then cooled to room temperature, followed by filtration using Whatman^®^ (US reference) # 1 filter papers. The concentration of the extract in water was determined to be 0.4562 g. The filtrate was kept in a refrigerated environment until it was needed.

### 2.3. Preparation of Chitosan-CuO Nanocomposite

**Figure 1:**
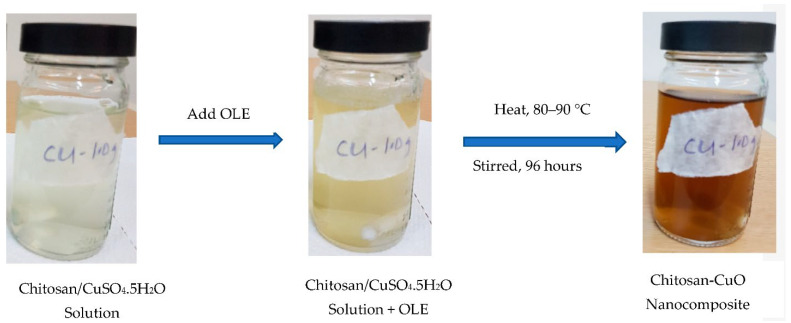
Schematic representation of biogenic in situ preparation of CH−CuO nanocomposite.

Chitosan samples, with a mass of 0.5, 1.0 and 2.0 g, respectively, were weighed into a 250 mL capacity conical flask, and 100 mL distilled H_2_O containing 1 mL of acetic acid was added. The mixture was agitated with a magnetic stirrer at room temperature until the chitosan was totally dissolved. An appropriate amount of copper sulphate pentahydrate (CuSO_4_⋅5H_2_O) (0.2497 g, 1 mM equivalent) was added to the chitosan solution and agitated. This was followed by the introduction of 5 mL of already made OLE. The mixture was heated using a magnetic hot plate at a constant temperature of 90 °C and continuously stirred for 96 h. The production of chitosan-CuO nanocomposite was detected by a gradual change in colour (Figure 1), which was validated by UV-vis measurement.

### 2.4. Characterization

An ATR-FTIR spectrophotometer (Nicolet iS5, Thermo Scientific, Waltham, MA, USA) covering the 4000 to 400 cm^−1^ range was used to characterize the produced CHT-CuO nanocomposite. The OLE and nanocomposite samples for FTIR analysis were prepared by placing a few drops of liquid samples in a Petri dish, which were evaporated to dryness in an oven at 40 °C and the solid residue was used for FTIR analysis. The UV-vis spectra of the CHT-CuO OLE-mediated nanocomposite was acquired by employing a JASCO770-UV–vis spectrophotometer (Tokyo, Japan). The apparatus was run at a 200 nm min^−1^ scan rate with a 1 nm resolution.

The colloidal solution of chitosan-copper oxide nanocomposite was centrifuged for 25 min at 10,230 rpm. After the treatment, the solid residues obtained from this method were rinsed three times with deionized H_2_O. The residues were redissolved in 100% ethanol and oven-dried at 50 °C, and XRD analysis was performed on the powder sample. Using nickel-filtered Cu K radiation = 1.5406 oA at 40 kV and 30 mA, the powder X-ray diffraction pattern was recorded using a Rigaku MiniFlex II (Tokyo, Japan) instrument.

TEM analysis was realized using a JEOL JEM-2100F instrument (Tokyo, Japan) to determine the size and shape of CuO nanoparticles in the composite. A small amount of CHT-CuO nanocomposite solution was placed on a Cu sample holder (carbon-coated), dried at ambient temperature, and imaged at 200 kV accelerating voltage.

The prepared CHT-CuO nanocomposite’s dynamic light-scattering analysis and zeta potential was measured utilizing a Malvern zetasizer ver. 7.12 (UK). A 1 M HCl or 1 M NaOH solution was used to alter the pH.

A LYRA3 TESCAN apparatus was used to conduct an FE-SEM investigation. The sample was made by placing a small amount of the nanocomposite solution on a sample holder made of Al, which was dried at room temperature to form thin films of the sample on the sample holder’s surface. The sample was subjected to FE-SEM analysis utilizing 20 kV accelerating voltage.

An energy-dispersive spectroscope (EDS), which was connected to the FE-SEM apparatus, was utilized to elucidate the elemental composition of the sample.

### 2.5. Bacteria Cultures

Cryopreserved bacterial cultures were resuscitated by 3 days of incubation in rich Luria broth medium (LB) at 37 degrees Celsius and 120 revolutions per minute. For determination of growth retardation in liquid culture (Section 2.7), bacteria were grown in LB medium for one day at 37 °C, 120 rpm, in the presence of the produced nanocomposite as antibacterial agents, and their growth was visually and quantitatively evaluated [47]. Solid agar plate cultures for “cup plating” were incubated for 12 h (Section 2.6), whereas bacterial enumeration was incubated for a day (Section 2.7). All of the tests were performed twice, and the average value was calculated.

### 2.6. Antibacterial Activity by ‘Cup Plating’ or “Disc Diffusion” Technique

The gross inhibiting activity of the chitosan-CuO nanocomposite was determined using the ‘cup and plating’ protocol published elsewhere [47,48]. Briefly, the approach is based on the use of a solid agar plate made in rich medium (Luria Broth, LB), on which is streaked around 100 microlitres of a fresh bacterial culture of approximately 10^6^ CFU/mL. Then, a 5 mm diameter by 2 mm height hole was made and filled with 100 µL of the antimicrobial chitosan-CuO nanocomposite. The chitosan-CuO nanocomposite then diffused around the hole, creating a zone of inhibition of bacterial growth (disc diffusion), the diameter of which was measured 12 h after incubation at 37 °C. The greater the diameter of this zone, the higher the inhibition effect of the prepared chitosan-CuO nanocomposite [48]. The antibiotics amoxicillin and ciprofloxacin were employed as known inhibitors at 10 and 50 mg/mL, respectively.

### 2.7. Determination of the Minimum Inhibitory Concentration (MIC) and Minimum Bactericidal Concentration (MBC)

Bacterial growth was monitored visually in LB liquid culture at 37 °C for 24 h in the presence of varied nanocomposite concentrations (10^−1^–10^–5^%), and growth was assessed visually by observing the culture turbidity, which indicates the presence of both active and lifeless bacteria [49]. The bacteriostatic effect or the lowest antimicrobial agent concentration in which turbidity is not detected was determined from the culture. According to this method, both viable and non-viable bacterial cells were used to calculate the MIC.

In the assessment of only viable bacterial cells, 100 microlitres of culture was placed on an agar LB plate and incubated at 37 °C for 12 h (after visual monitoring, as indicated in the preceding section). Only live bacteria can grow and produce colony-forming units (CFUs) on an agar plate. If the bacterial concentration in the original liquid media was high, the culture was diluted (by a factor of 10 for five dilutions) before being transferred to the solid plate. The lowest concentration of nanocomposite that limited bacterial growth was designated as MBC, and viable bacteria were counted as CFUs. The negative control in these trials was culture medium that was free of micro-organisms and antimicrobial agents. The goal of these checks was to make sure the culture medium was not contaminated by micro-organisms. Contrarily, the positive control, comprised of bacterial cultures devoid of nanocomposite, was used to determine the bacteria’s maximal growth rate. In this investigation, the following bacteria were examined: *Bacillus licheniformis*, *Staphylococcus haemolyticus*, *Bacillus cereus*, and *Micrococcus luteus* (Gram-positive), as well as *Pseudomonas aeruginosa*, *Pseudomonas citronellolis*, *E. coli*, *Klebisiella* sp., *Bradyrhizobium japonicum*, and *Ralstonia pickettii* (Gram-negative).

## 3. Results and discussion

### 3.1. Synthesis of Chitosan-CuO Nanocomposite

The bio-inspired technique was used to synthesize CHT-CuO nanocomposite utilizing OLE. The synthesis of the CHT-CuO nanocomposite was initially confirmed by the change of colour from colourless (chitosan-CuO solution) (due to low concentration of the precursor used) to yellowish colour with the addition of OLE and eventually to dark-brown colour, signalling the formation of CHT-CuO nanocomposite, as illustrated in Figure 1. For the creation of CHT-CuO nanocomposite, OLE worked as a reducing agent, and chitosan molecules may have played a major role as stabilizing and capping agents [50]. Olive leaf water extract is expected to contain secoiridoids, such as oleuropein, ligstroside, 1-methyloleuropein, and oleoside; flavanoids, such as apigenin, kaempferol, luteolin, and chrysoeriol; and phenolic compounds, such as caffeic acid, tyrosol, and hydroxytyrosol, in agreement with reports in the literature [51]. These phytoconstituents could have played an important role in the reduction process. 

### 3.2. Analysis of UV–Visible Spectroscopy

**Figure 2:**
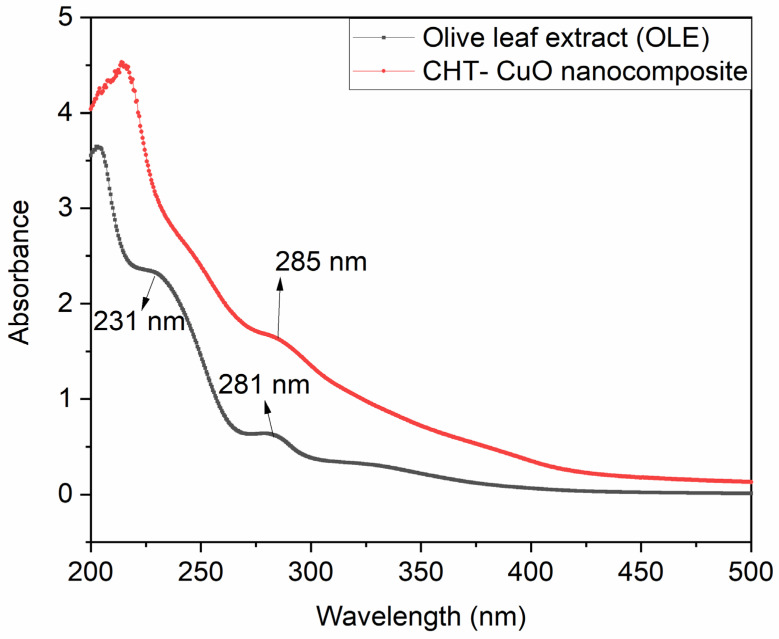
UV-vis spectra of OLE and CHT−CuO nanocomposite.

UV-vis is a great tool for recognizing, characterizing, and researching nanomaterials because nanoparticles have distinctive optical characteristics that are sensitive to size, shape, concentration, aggregation state, and refractive index near the nanoparticle surface, generally attributed to the so-called surface plasmon resonance (SPR) effect. UV-visible spectroscopy (UV-vis) analyses the attenuation of light (scattered or absorbed) passing through a substance. UV-visible spectroscopy was used to monitor the synthesis of the CHT-CuO nanocomposite utilizing OLE. CuO nanoparticles have a pronounced blue shift in their UV absorption spectra in comparison to bulk CuO. CuO nanoparticles show a high density of surface defects, interstitials, and oxygen vacancies due to their high surface-area-to-volume ratio. In the current study, the spectral absorbance peak of the prepared CHT-CuO nanocomposite shifted around 285 nm (Figure 2). This wavelength is lower than the value of 316 nm reported by Bharathi et al. [37]. The difference in the peak position could be attributed to many factors, such as the influence of particle size and shape, the dosage of chitosan, the local refractive index, and methods of preparation, amongst others. Figure 2 also depicts the UV-vis spectrum of the extract (OLE) alone for comparison purposes. The spectrum of OLE exhibited two peaks at 231 and 281 nm. Neither of these peaks is observed in the spectrum of the chitosan-CuO nanocomposite, indicating the successful conversion of copper sulphate to copper oxide utilizing some of the phytoconstituents present in OLE. 

### 3.3. FTIR Analysis

**Figure 3:**
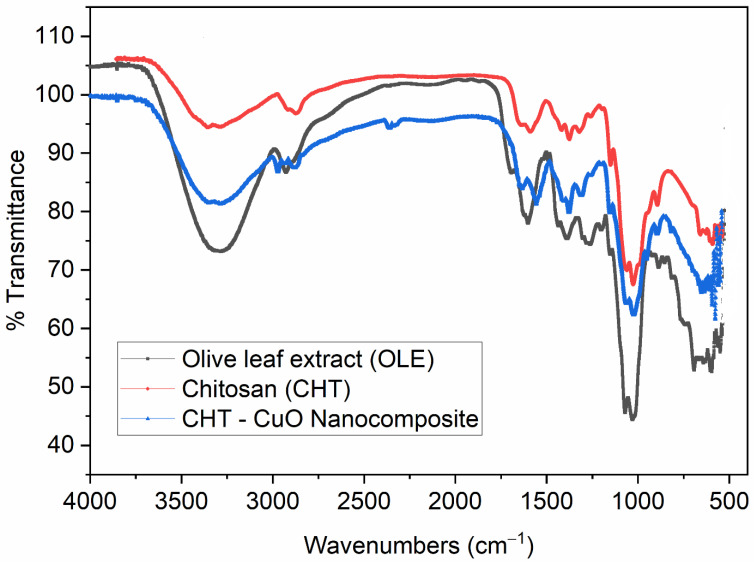
FTIR spectra of OLE, chitosan, and CHT− CuO nanocomposite.

FTIR spectra of OLE, chitosan, and chitosan-CuO nanocomposite samples are depicted in Figure 3. FTIR spectra of the chitosan-CuO nanocomposite sample reveal important peaks at 3286, 2971, 2879, 2358, 1557, 1378, 1152, 1022, 895, and 577 cm^−1^. The bands at 3286 cm^−1^ could be attributed to O-H stretching, indicating the presence of hydroxyl groups, whereas the peak at 2879 cm^−1^ is assigned to C-H stretching. Peaks at 1634 cm^−1^ and 1022 cm^−1^ show N-H bending and C-N stretching of amine groups, respectively. The bands at 1557 and 1153 cm^−1^ may be attributed to C=C stretching and strong C-O stretching, respectively. Peaks observed in the range of 593–637 cm^−1^ are attributed to the metal–oxygen bond, which could be due to CuO in the nanocomposite [52]. A similar observation was previously reported [53]. The FTIR spectrum of chitosan is presented in Figure 3, showing characteristic peaks at 3358, 2873, 1589, 1375, 1149, 1026, 893, and 592 cm^−1^. The absorption bands in the vicinity of 1149 and 1028 cm^−1^ correspond to typical saccharide moiety characteristic peaks [36], but the peak at 2877 cm^−1^ corresponds to the –CH stretching vibration. The band at 3358 cm^−1^ is assigned to −OH and −NH_2_ stretching vibrations, whereas the separate peaks at 1674 and 1589 cm^−1^ are related to –NH_2_ bending vibrations, and the peak at 1375 cm^−1^ is attributed to amide III: C-N stretching vibrations.

The OLE FTIR spectrum (Figure 3) exhibited distinguished peaks at 3285, 2927, 1602, 1391, 1261, 1070, 1030, 692, and 549 cm^−1^. The intense peak in the vicinity of 3285 cm^−1^ in the spectrum is typical of the O–H stretching of polyphenols [54]. The intense bands at 2927 cm^−1^ correspond to −CH stretching, which may come from the phytoconstituent of bioactive compounds present in OLE [55]. The distinct band at 1602 cm^−1^ is attributed to -NH bending, indicating the presence of -NH bending of amine. The characteristic peak at 1261 cm^−1^ is also attributed to strong C-O stretching, indicating the presence of a carboxylate group. The presence of bioactive phytochemicals, such as triterpenes, proteins, steroids, carbohydrates, alkaloids, and other substances, in the OLE may cause unsaturated C-H bending and C-N stretching between 1152 and 1000 cm^−1^. These chemicals may have a capping effect, which contributes to the stability of the chitosan-CuO nanocomposite. 

### 3.4. Studies on the Zeta Potential (ZP)/Dynamic Light Scattering (DLS) Analysis

**Figure 4:**
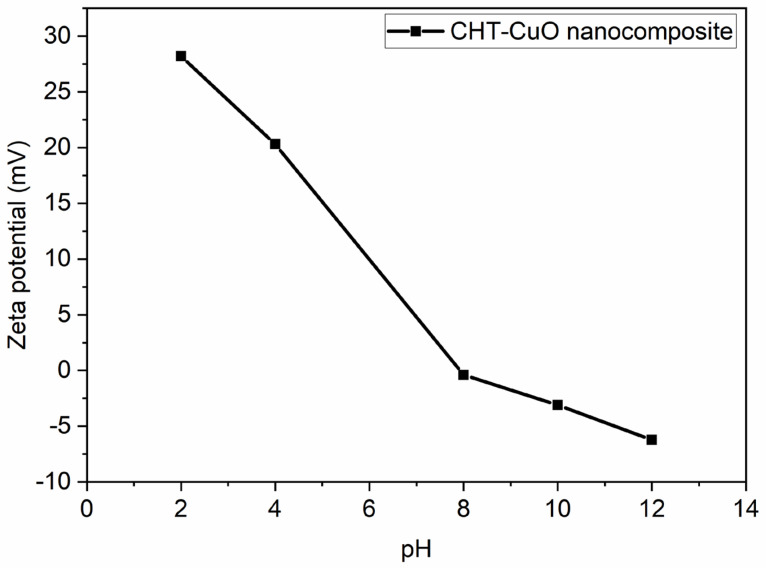
Zeta potential plot for CHT− CuO nanocomposite at different pH values.

The ZP value was utilized to determine nanoparticle colloidal stability [56,57,58]. The following categorization was adopted for ZP: A colloidal solution with a ZP value in the range of 0–10 mV was considered unstable. A ZP value of 10–20 mV or ± 20–30 mV indicates a relatively or moderately stable colloid, respectively. The ZP value for a very stable nanoparticle colloid is predicted to be higher than 30 mV [56,57]. The ZP value for the chitosan-CuO nanocomposite in the present work (Figure 4) is +28.2 mV under an acidic pH of 2. These results imply that the tested nanocomposite is moderately stable. The greater ZP value of the OLE-mediated chitosan-CuO nanocomposite demonstrates the influence of the reducing agent on nanoparticle formation and stability. 

The pH of a sample in aqueous conditions is one of the most critical elements affecting its ZP. Consider a particle suspended in a liquid with a negative zeta potential. When a large quantity of alkali is introduced to the suspension, the particles become more negatively charged. If acid is introduced to the suspension, it reaches a point where the charge is neutralized. If the ions are specifically adsorbed, adding more acid may induce a build-up of positive charge. A zeta potential versus pH curve in this situation would be positive under lower pH and lower or negative under greater pH. The isoelectric point is when the graph passes through zero ZP and is particularly essential from a practical standpoint. It is usually where aggregation is most frequent and the colloidal system is the least stable. Figure 4 depicts a typical zeta potential vs. pH curve. The isoelectric point of the sample can be seen on the plot to be around pH 7.5. The figure can also be utilized to estimate that the sample will be stable at pH values lower than 4 (more positive charge) and greater than pH 7.5. (more negative charge). Because the ZP values are between +30 and −30 mV [58,59,60], problems with dispersion stability are likely for pH levels between 6 and 12.

For particle size analysis in the nanoscale range, dynamic light scattering is a well-established, standardized approach. DLS gives information on particle size distribution, as well as mean particle size. Figure S1 (Supplementary Information) shows the particle size distribution of CuO produced using the DLS approach. The average particle size is roughly 50 nm, as shown in the diagram. This value is higher than what TEM measurements revealed. There have been some proposed explanations for such disparities. TEM measurement is performed in the dry state, whereas DLS measurement is performed in the solvated condition. Furthermore, TEM is a number-based particle size measurement that places a greater emphasis on the smallest components of the size distribution, whereas DLS is an intensity-based measurement that places a greater emphasis on larger particle sizes.

### 3.5. XRD Studies

**Figure 5:**
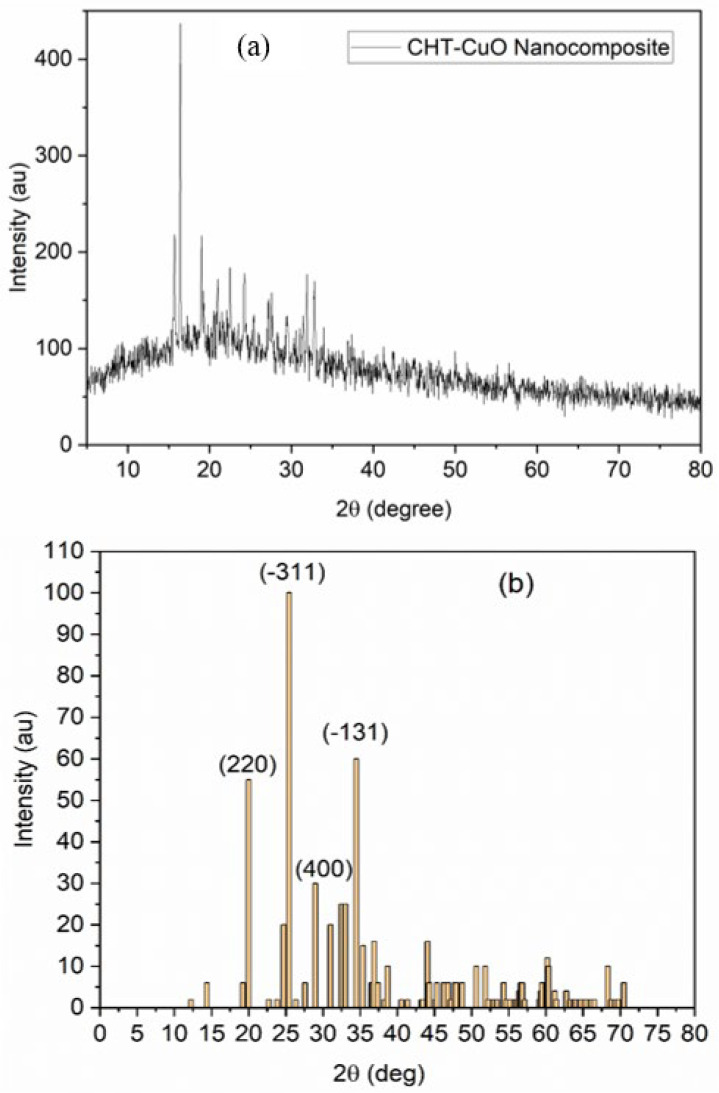
(**a**) XRD pattern and (**b**) XRD peak plots for CHT−CuO nanocomposites.

Figure 5a shows the CuO NP XRD pattern in the composite formed by treating 5 mL of OLE with 1.0 g chitosan + 1 mM aqueous CuSO_4_⋅5H_2_O solution, and Figure 5b shows the obtained diffraction peaks (b). The crystalline structure, crystalline grain size, phase nature, lattice parameter, and amorphous nature of the nanocomposite were all investigated using XRD [61]. Near 2-Theta = 19.98°, 25.43°, 28.98°, and 34.38°, prominent peaks can be seen. These peaks correspond to orientation planes (220), (−311), (400), and (−113), respectively, which are indexed to a typical monoclinic structure and benchmarked with the Joint Committee on Powder Diffraction Standards (JCPDS Card No. 01-085-1693). Some of the peaks, particularly those at 25.43° and 34.38°, which correspond to (−311) and (−113) orientations, respectively, are outside of known CuO nanoparticle diffraction peaks. The existence of contaminants in the produced nanocomposite could explain these peaks [47]. Mineral components in plant-based materials that covered the surface of the nanoparticles [47] or unused copper sulphate could be the source of the contaminants.

### 3.6. FESEM/EDS Analysis

**Figure 6:**
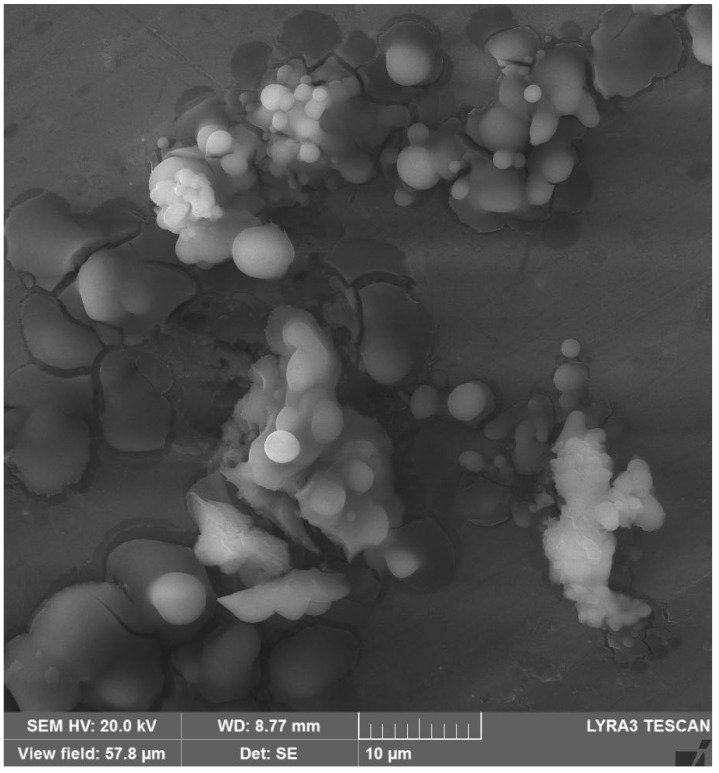
FE-SEM image of CHT−CuO nanocomposite.

**Figure 7:**
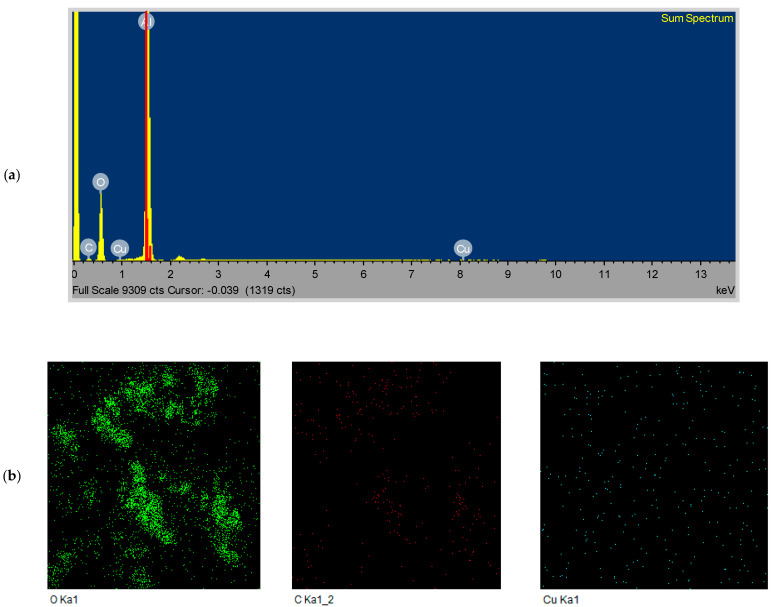
(**a**) EDS spectrum and (**b**) elemental mapping of CHT−CuO nanocomposite.

FE-SEM images of the prepared chitosan-CuO nanocomposite are presented in Figure 6, corresponding EDS images are depicted in Figure 7a, and elemental mapping is presented in Figure 7b. As can be clearly seen in Figure 6, OLE successfully reduced CuSO_4_⋅5H_2_O to CuO nanoparticles, which are spherical in shape and polydispersed. In the corresponding EDS images (Figure 7a,b), typical optical absorption peaks of metallic Cu nanocrystals at 1 and 8 keV can be clearly observed (Figure 7a), thus validating the formation of CuO NPs. Figure 7b shows the elemental mapping of the chitosan-CuO nanocomposite. EDS revealed a high-intensity metallic peak of elements such as aluminium (Al), copper (Cu), oxygen (O) and low-intensity peaks of carbon (C) [62]; their elemental mapping is clearly shown (Figure 7b). The high-intensity peak of Al emanates from the sample holder. For CuO nanoparticles (NPs), EDS analysis revealed a fairly homogeneous copper-rich composition (Figure 7b). The chitosan that surrounds the CuO NPs may be responsible for the carbon and oxygen signals [63,64]. 

The XRD, SEM, TEM, and DLS analysis results were similar for all the nanocomposites prepared in the presence of varying amounts of chitosan. This suggests that the concentration of chitosan did not influence the quality of nanoparticles formed.

### 3.7. TEM Analysis

**Figure 8:**
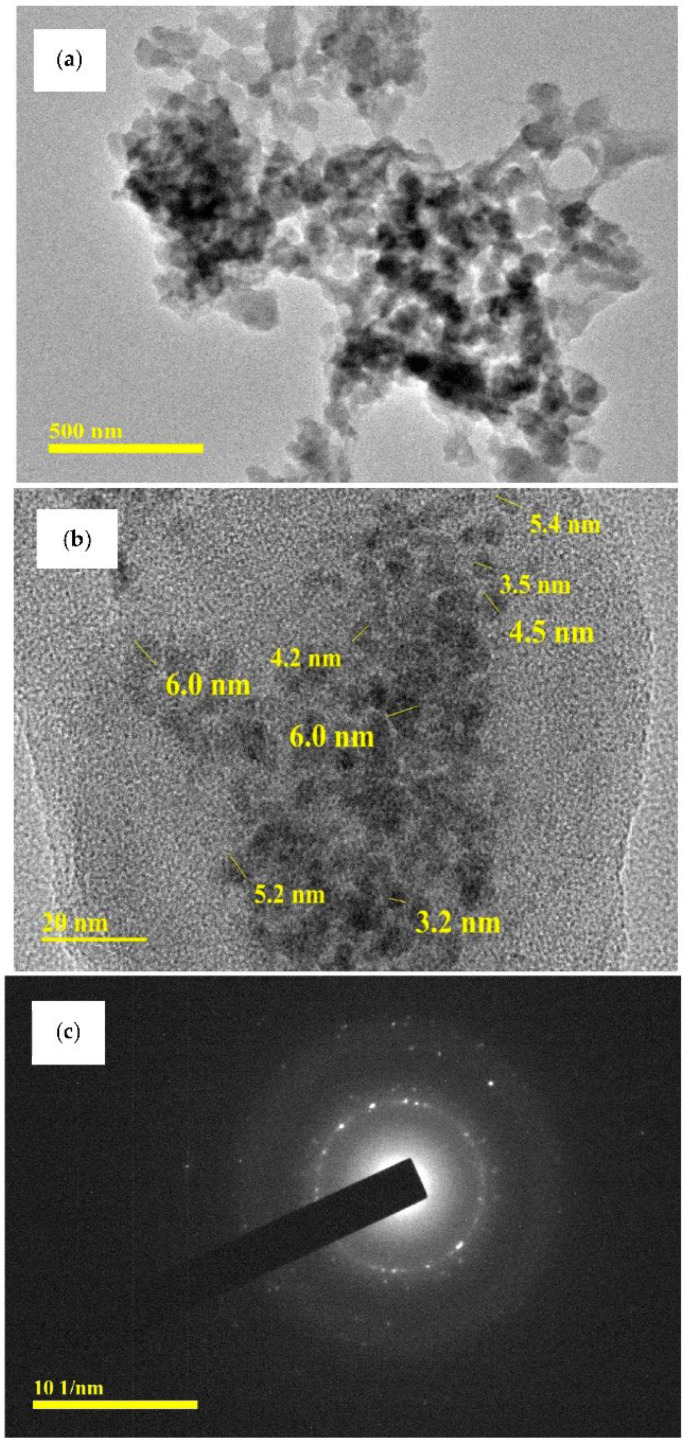
(**a**,**b**) TEM micrographs of CHT-CuO nanocomposite at different magnifications and (**c**) SAED pattern.

TEM examination was used to identify the shapes and sizes of the biosynthesized chitosan-CuO nanocomposite. TEM pictures at 500 nm and 20 nm magnifications are shown in Figure 8a,b. Figure 8c depicts the SAED (selected area electron diffraction) patterns of the chitosan-CuO nanocomposite at a magnification of 101 nm. The tested chitosan-CuO nanocomposite is polydispersed, spherical, and of various sizes. In an OLE-mediated nanocomposite, the size of the CuO NPs is in the range of 3.2–6.0 nm (Figure 8b). Previously, several researchers [62] related size diversity to formation time variations. According to the SAED pattern, the CuO NPs are embedded in the chitosan matrix. The SAED pattern further reveals that the CuO nanoparticles are polycrystalline [62], as the diffraction point (Figure 8c) is dispersed on concentric rings. The brilliant circular spots found in the SAED patterns in Figure 8c support the crystalline character of the nanoparticles indicated by the XRD results (Figure 5a).

### 3.8. Antimicrobial Studies

**Figure 9:**
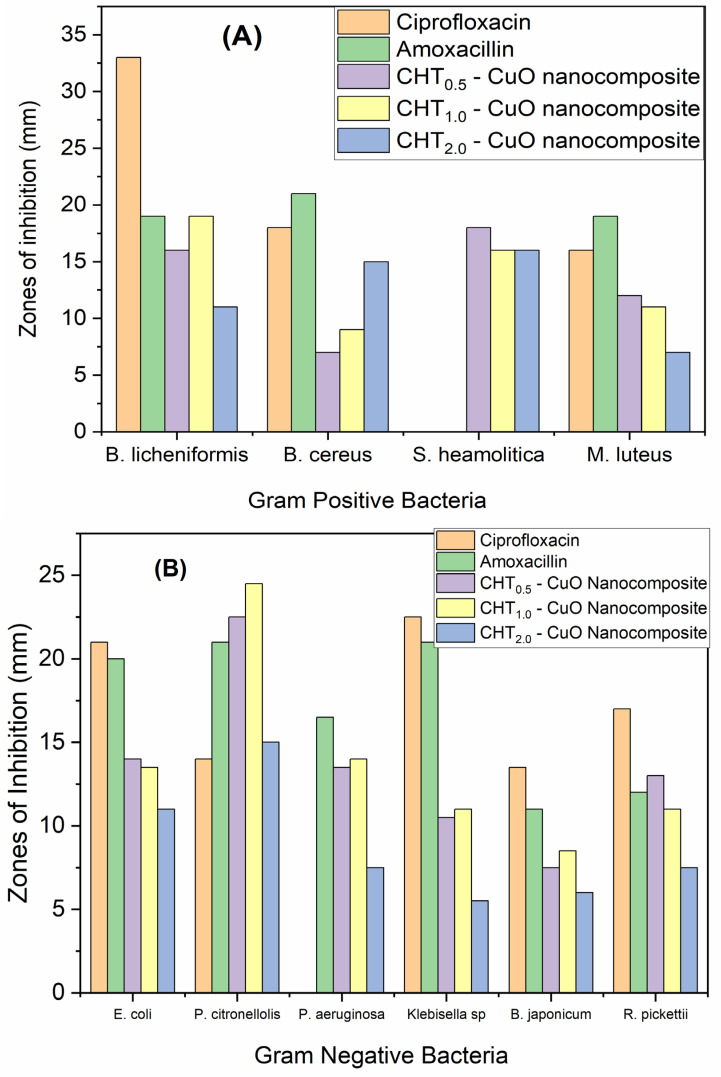
Zones of inhibition showing antibacterial activity of the chitosan-CuO nanocomposite in comparison with antibiotics (ciprofloxacin and amoxicillin) against (**A**) Gram-positive and (**B**) Gram-negative bacteria.

The antimicrobial activities of the synthesized nanocomposites, using different amounts of chitosan (0.5 [CuO-0.5], 1.0 [CuO-1] and 2.0 g [CuO-2]) were studied using the cup-platting (or disc diffusion) technique against Gram-positive bacteria (*B. licheniformis*, *S. haemolyticus*, *B. cereus*, and *M. luteus*) and Gram-negative bacteria (*P. aeruginosa*, *P. citronellolis*, *E. coli*, *Klebisiella* sp., *B. japonicum*, and *R. pickettii*) (Figure 9, Figures S2 and S3) [46]. The known antibiotics ciprofloxacin and amoxicillin were employed for comparison. 

Overall, against all tested bacterial strains, the diameters of the inhibition of the three nanocomposites fell between 6 and 24 mm, and no noticeable difference was observed between Gram-negative and Gram-positive bacteria (Figure 9a,b). Figures S2 and S3 provide pictures of the diameter of inhibition of each tested bacteria. Interestingly, nanocomposites made with 2.0 g of chitosan (CuO-2) were associated with the smallest diameters of the inhibition zone against the tested bacteria (except in *B.* *cereus*), reflecting their lower antimicrobial activities. The order of the antimicrobial activity was CuO-1 > CuO-0.5 > CuO-2. An inhibition zone greater than 1 mm indicates satisfactory antibacterial potential on the basis of the SNV 195920-1992 Standard Antibacterial Test [65,66,67].

The reference antibiotics, amoxicillin and ciprofloxacin, showed higher antimicrobial activity, with a diameter of inhibition zone falling between 15 and 32 cm. However, it is important to note that these reference antibiotics were less active than nanocomposites in three bacterial strains. For instance, the two tested antibiotics did not show an inhibition zone against *S. Heamolityca*; likewise, no inhibition zone was observed with the use of ciprofloxacin against *P. aeruginosa*. Interestingly, against *P. citronellolis*, the two tested antibiotics were less active than the three nanocomposites, illustrating the potential of this new nanocomposite material against some bacterial strains. 

Further assessment of the nanocomposite’s antibacterial effect was performed in a liquid medium. As shown in Table S1, complete inhibition of bacterial growth was observed at a concentration of 0.1–0.01% of the nanocomposite (Supplementary Information). Furthermore, as expected, as the dilution of the nanocomposite increases, the growth inhibition decreases (Table S1). The use of nanocomposites at 0.1% (irrespective of chitosan concentration) inhibited all tested bacteria, except *S. heamoylitica*. At 0.01%, the nanocomposite CuO-0.5 inhibited more bacterial strains than CuO-2 (mainly Gram-negative bacteria, Table S1). Overall, the nanocomposites CuO-0.5 and CuO-1 were more active than CuO-2. 

Table 1 summarizes MIC and MBC of the nanocomposites. MIC is the lowest concentration at which turbidity is visible, and this turbidity is associated with both with viable and dead bacteria, whereas MBC is associated with viable bacteria only. Overall, against each bacterial strain, MIC and MBC values of the three nanocomposites were identical, except in *M. luteus*. In this strain, the three nanocomposites have MIC and MBC values of 0.01 and 0.1%, respectively (Table 1). The fact that MIC and MBC are the same suggests that the turbidity of the cultures was mostly caused by live bacteria. Thus, in the case of *M*. *luteus*, viable and non-viable cells account for this turbidity. 

Interestingly, the nanocomposite MIC/MBC values were > 0.1% against *S. heamolitica*, indicating this strain is less susceptible to these antibacterial agents. Based on the disc diffusion experiment (Figures S2 and S3), this strain was not inhibited by the reference antibiotics ciprofloxacin and amoxicillin (at 10 mg/L), whereas the inhibition zone was observed with the nanocomposites (Figures S2 and S3). This indicates that nanocomposites could inhibit these strains at concentrations higher than 0.1%. Another interesting information is that against Gram-negative bacteria, the nanocomposites CuO-0.5 and CuO-1.0 have MIC/MBC values of 0.01%, whereas values for CuO-2 were 0.1% against *Klebisella* sp., *B. japonicum*, and *R. pickettii* and 0.01% against the two tested *Pseudomonas* species (*P. citronellolis* and *P. aeruginosa*). The only exception was *E. coli*, against which CuO-2 was more active than the other two tested nanocomposites. Thus, overall, CuO-0.5 and CuO-1.0 were more active than CuO-2, which is in line with the observations from the disc diffusion experiment (Figures S2 and S3 and Figure 9). Furthermore, these findings revealed that Gram-positive bacteria were less vulnerable to nanocomposites than Gram-negative bacteria. The strong antibacterial activity demonstrated by OLE-mediated chitosan-CuO nanocomposites can be attributed to the release of CuO NPs, which kill bacteria by any or all of the methods previously mentioned. Low-molecular-weight chitosan has also been shown to have antibacterial properties. Costa et al. [68] speculate that the action emanates from chitosan’s capacity to commune with and destroy micro-organism cell walls via hole formation or membrane breakdown. 

In our previous studies, we reported that chitosan exhibited antibacterial activity against the tested bacteria strains [46]. In order to assess the origin of the observed antibacterial activity, olive leaf extract (OLE) alone was also tested. The obtained results are depicted in Table 2 and Figure S4. Plant extracts (OLE) had no inhibitory effect against eight (8) of the studied bacteria, with little inhibition of growth of *P. aeruginosa* and *B. japonicum*, with inhibition zone diameters of 3.5 ± 0.0 mm and 2.0 ± 0.0 mm, respectively. Many reports in the literature have also shown that CuO nanoparticles exhibit excellent antibacterial activity [39,40,41,42]. It is therefore pertinent that the combined action of chitosan and CuO nanoparticles could be responsible for the excellent antibacterial action of OLE-mediated chitosan-CuO nanocomposites.

Antibacterial activity increases when the concentration of chitosan increases from 0.5 g to 1.0 g; however, increasing the concentration to 2.0 g resulted in a decline in antibacterial activity for the majority of the bacteria strains examined. Similar findings have been reported in the literature, where cotton fabrics treated with chitosan concentrations of 0.5–0.75% showed the highest antibacterial activity and increasing the chitosan content to 1% resulted in a decrease in antibacterial activity [69]. The possible reasons for this, according to the literature report, are as follows: when the concentration of chitosan is lower, chitosan combines with the surface of bacteria cells with a negative charge, disrupts the membrane of bacteria cells, and induces component leakage in bacteria cells, eventually leading to the death of bacteria cells. When the concentration of chitosan is higher, however, protonated chitosan can be wrapped around the surface of bacterial cells to prevent component leakage, and positively charged bacterial cells repel each other to avoid agglutination [70].

The findings reveal that the OLE-mediated chitosan-CuO nanocomposite is particularly effective against the bacteria tested, which is consistent with previous studies on the antimicrobial activity of natural polymers/CuO nanocomposites [66,67]. The nanocomposite’s activity could be attributed to its small size and capacity to increase bacteria’s surface contact, perhaps inflicting injury. The bactericidal mechanism of metal and metal oxide nanocomposites is based on the formation of reactive oxygen species, such as superoxide radical anions, hydrogen peroxide anions, and hydrogen peroxide, which commune with bacterial cell walls, giving rise to the destruction of the cell membrane, inhibiting further cell growth, and causing leakage of internal cellular components, ultimately leading to bacterial death [43]. Reactive oxygen species created by metal oxide nanocomposites obstruct bacterial cell processes, such as glutathione depletion, DNA rupture, protein denaturation, and enzyme damage, preventing bacterial cell reproduction.

## 4. Conclusions

The green synthesis, characterisation, and antibacterial efficacy of chitosan-CuO nanocomposites made with olive leaves extract (OLE) as the reducing agent were described in this paper. FTIR, UV-vis, XRD, FE-SEM, and TEM were used to confirm the effective synthesis of an OLE-mediated chitosan-CuO nanocomposite. XRD, SEM, TEM, and DLS analysis results were similar for all the nanocomposites prepared in the presence of varying amounts of chitosan. This suggests that the concentration of chitosan did not influence the quality of nanoparticles formed. The CuO nanoparticles in the composite are very stable and polydispersed. The CHT-CuO nanocomposite’s zeta potential varies with pH. The largest zeta potential (+28 mV) was found at pH 2, whereas the lowest (−7.5 mV) was found at pH 12. According to the FTIR data, the loose carboxylate functional groups in the plant-based material (extract) stabilize the nanoparticles in the polymer matrix. OLE works well as a reducing agent, resulting in smaller nanoparticles with sizes in the range of 3.2–6.0 nm based on TEM analysis. These values are lower than those obtained from DLS analysis, which were found to be around 50 nm. Gram-positive bacteria, including *B. licheniformis*, *B. cereus*, and *M. luteus*, as well as Gram-negative bacteria, such as *E. coli*, *P. citronellolis*, *P. aeruginosa*, *kliebisella* sp., *Bradyrhizobium japonicum*, and *Ralstonia pickettii*, are successfully inhibited by OLE-mediated chitosan-CuO nanocomposites. Commercially available antibiotics (ciprofloxacin and amoxicillin) had little effect on *Staphylococcus haemolytica*, but an OLE-mediated chitosan-CuO nanocomposite did. The plant extracts (OLE) had no inhibitory effect against eight (8) of the studied bacteria, with little inhibition of growth of *P. aeruginosa* and *B. japonicum* with inhibition zone diameters of 3.5 0.0 mm, 2.0 and 0.0 mm, respectively. The smaller nanoparticles and capacity to overcome biological barriers may explain the greater antibacterial activity of OLE-mediated chitosan-CuO nanocomposites. It is pertinent that the antibacterial activity of the prepared nanocomposites could be largely attributed to the combined effect of chitosan and CuO nanoparticles. This environmentally friendly chitosan-CuO nanocomposite is a cost-effective, biogenic molecule capable of acting as an antibacterial agent against Gram-positive and Gram-negative bacteria.

## Figures and Tables

**Table 1:** Minimum inhibitory concentration that inhibits bacteria growth based on visual turbidity or bacteriostatic effect (MIC, in %) and minimum inhibitory concentration that inhibit growth in solid plate culture or bactericidal effect (MBC, in %). For CHT-CuO nanocomposites.

Type	Bacteria	CHT_0.5_-CuO Nanocomposite (%)		CHT_1.0_-CuO Nanocomposite (%)		CHT_2.0_-CuO Nanocomposite (%)	
Gram-positive		**MIC**	**MBC**	**MIC**	**MBC**	**MIC**	**MBC**
	*B. licheniformis*	0.1	0.1	0.1	0.1	0.1	0.1
	*B. cereus*	0.01	0.01	0.01	0.01	0.01	0.01
	*S. heamolytica*	>0.1	>0.1	>0.1	>0.1	>0.1	>0.1
	*M. luteus*	0.01	0.1	0.01	0.1	0.01	0.1
Gram-negative	*E. coli*	0.1	0.1	0.1	0.1	0.01	0.01
	*P. citronellolis*	0.01	0.01	0.01	0.01	0.01	0.01
	*P. aeruginosa*	0.01	0.01	0.01	0.01	0.01	0.01
	*Klebsiella* sp.	0.01	0.01	0.01	0.01	0.1	0.1
	*B. japonicum*	0.01	0.01	0.01	0.01	0.1	0.1
	*R. pickettii*	0.01	0.01	0.01	0.01	0.1	0.1

**Table 2:** Antimicrobial effect of olive leaf extract (OLE) using cup plate experiments. Values stand for the diameter (in mm) of zones of inhibition.

Type	Bacteria	Diameter of Zones of Inhibition (mm)		
		**1st Run**	**2nd Run**	**Average Values**
Gram-positive	*B. licheniformis*	0.0 ± 0.0	0.0 ± 0.0	0.0 ± 0.00
	*B. cereus*	0.0 ± 0.0	0.0 ± 0.0	0.0 ± 0.0
	*S. heamolitica*	0.0 ± 0.0	0.0 ± 0.0	0.0 ± 0.0
	*M. luteus*	0.0 ± 0.0	0.0 ± 0.0	0.0 ± 0.00
Gram-negative	*E. coli*	0.0 ± 0.0	0.0 ± 0.0	0.0 ± 0.0
	*P. citronellolis*	0.0 ± 0.0	0.0 ± 0.0	0.0 ± 0.0
	*P. aeruginosa*	3.0 ± 0.0	4.0 ± 0.0	3.5 ± 0.0
	*Klebsiella* sp.	0.0 ± 0.00	0.0 ± 0.0	0.0 ± 0.0
	*B. japonicum*	2.0 ± 0.0	2.0 ± 0.0	2.0 ± 0.0
	*R. pickettii*	0.0 ± 0.0	0.0 ± 0.0	0.0 ± 0.0

## Data Availability

The data presented in this study are available on request from the corresponding authors.

## Supplementary Materials

The following supporting information can be downloaded at: https://www.mdpi.com/article/10.3390/polym14091832/s1, Figure S1: Particle size distribution of CuO nanoparticles obtained by DLS method; Figure S2: Inhibition of bacterial growth by cup plate experiment. A, B, C and D show inhibition of *Bacillus licheniformies, Staphylococcus heamolitica*, *Bacillus cereus* and *Micrococcus luteus* respectively in the presence of 1- Ciprofloxacin, 2-Amoxacillin, 3-CHT_0.5_-CuO nanocomposite, 4- CHT_1.0_-CuO nanocomposite and 5- CHT_2.0_-CuO nanocomposite; Figure S3: Inhibition of bacterial growth by cup plate experiment. A, B, C, D, E and F show inhibition of *Escherichia coli*, *Pseudomonas citronellolis*, *Pseudomonas aeruginosa*, *Klebisella* sp., *Bradyrhizobium japonicum and Ralstonia pickettii* respectively in the presence of 1- Ciprofloxacin, 2-Amoxacillin, 3-CHT_0.5_-CuO nanocomposite, 4- CHT_1.0_-CuO nanocomposite and 5- CHT_2.0_-CuO nanocomposite; Figure S4: Inhibition of bacterial growth by cup plate experiment in the presence of OLE for all bacteria strains; Table S1: Assessment of the antimicrobial effect of CHT -CuO nanocomposites on gram positive and gram negative bacteria based on turbidity visualization of the culture and bacterial count on solid Agar plate. Values in brackets represent bacteria counts or colony forming units (CFU × 10^6^/mL) after 24 h culture. Positive control presents culture liquid without bacterial inhibitor substance, and negative control is the culture without bacteria. ‘–’ infers complete growth inhibition where ‘+’ indicate bacteria growth.
