# Supplementary material for: Biogenic Synthesis and Characterization of Chitosan-CuO Nanocomposite and Evaluation of Antibacterial Activity against Gram-Positive and -Negative Bacteria
Source: Polymers (Basel). 2022 Apr 29;14(9):1832. doi: 10.3390/polym14091832 (PMC9104765; doi:10.3390/polym14091832)
Supplement: Supplementary file 1 [file polymers-14-01832-s001.zip › polymers-1677242-supplementary.pdf]

ogenic synthesis and characterization of chitosan-CuO nanocomposite and evaluation of antibacterial activity against gram positive and negative bacteria

Peace S. Umoren[1], Doga Kavaz[1], Alexis Nzila[2], S. Saravanan[2], Saviour A. Umoren[3*]

[1] *Department of Bioengineering, Cyprus International University, Nicosia, North Cyprus*

[2] *Department of Bioengineering, King Fahd University of Petroleum and Minerals (KFUPM), Dhahran 31261, Saudi Arabia*

[3] *Interdisciplinary Research Center for Advanced Materials, King Fahd University of Petroleum and Minerals (KFUPM), Dhahran 31261, Saudi Arabia*

*For correspondence: umoren@kfupm.edu.sa (S. A. Umoren)

SUPPLEMENTARY MATERIALS

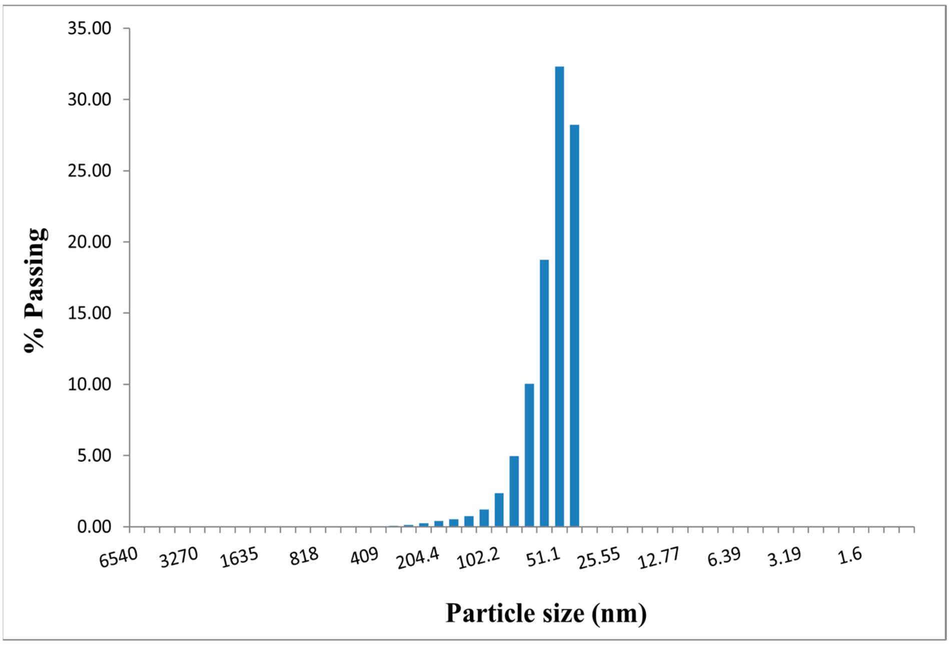

Figure S1: Particle size distribution of CuO nanoparticles obtained by DLS method

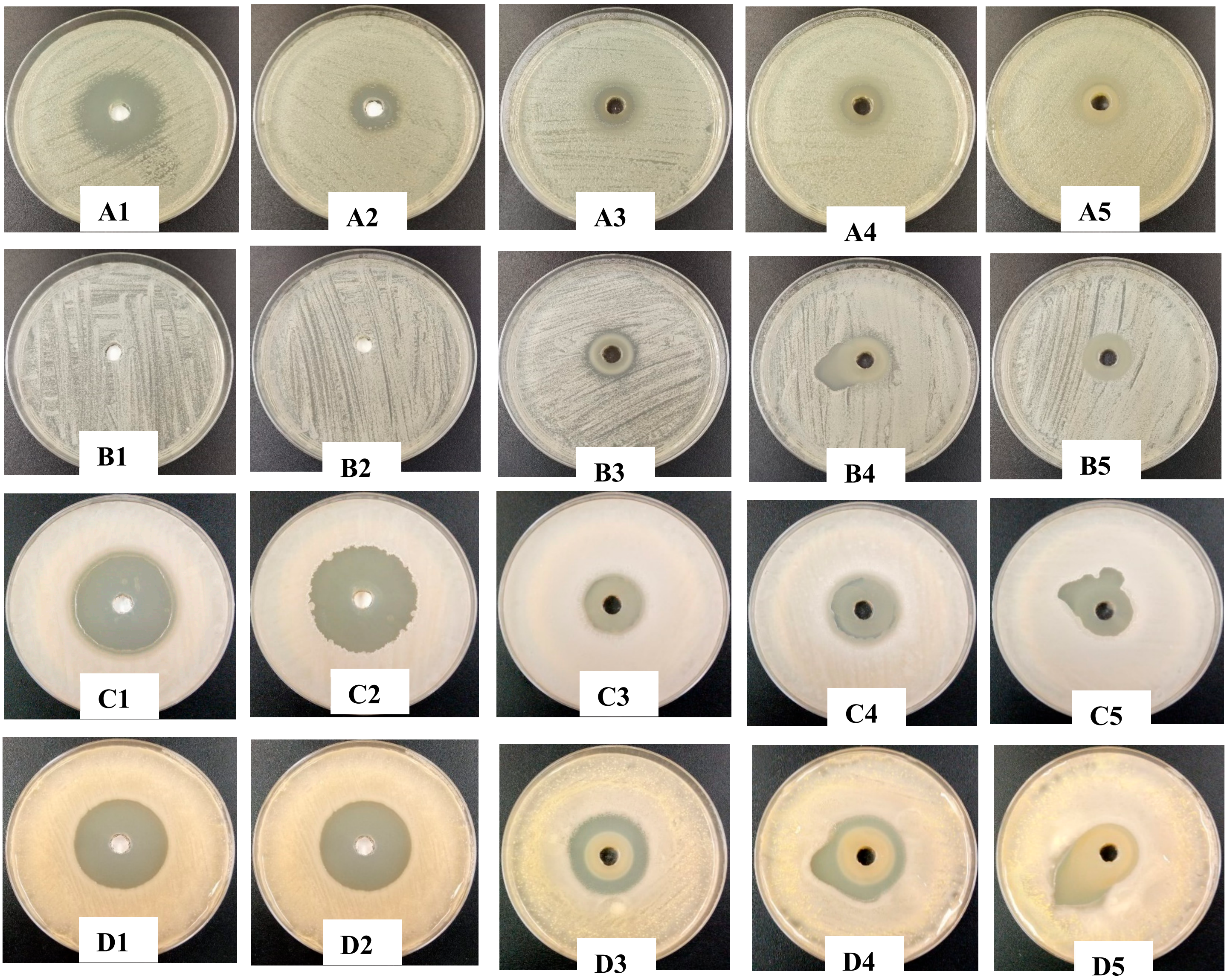

Figure S2: Inhibition of bacterial growth by cup plate experiment. A, B, C and D show inhibition of *Bacillus licheniformies, Staphylococcus heamolitica*, *Bacillus cereus* and *Micrococcus luteus* respectively in the presence of 1- Ciprofloxacin, 2-Amoxacillin, 3-$CHT_{0.5}$-CuO nanocomposite, 4 – $CHT_{1.0}$-CuO nanocomposite and 5- $CHT_{2.0}$-CuO nanocomposite

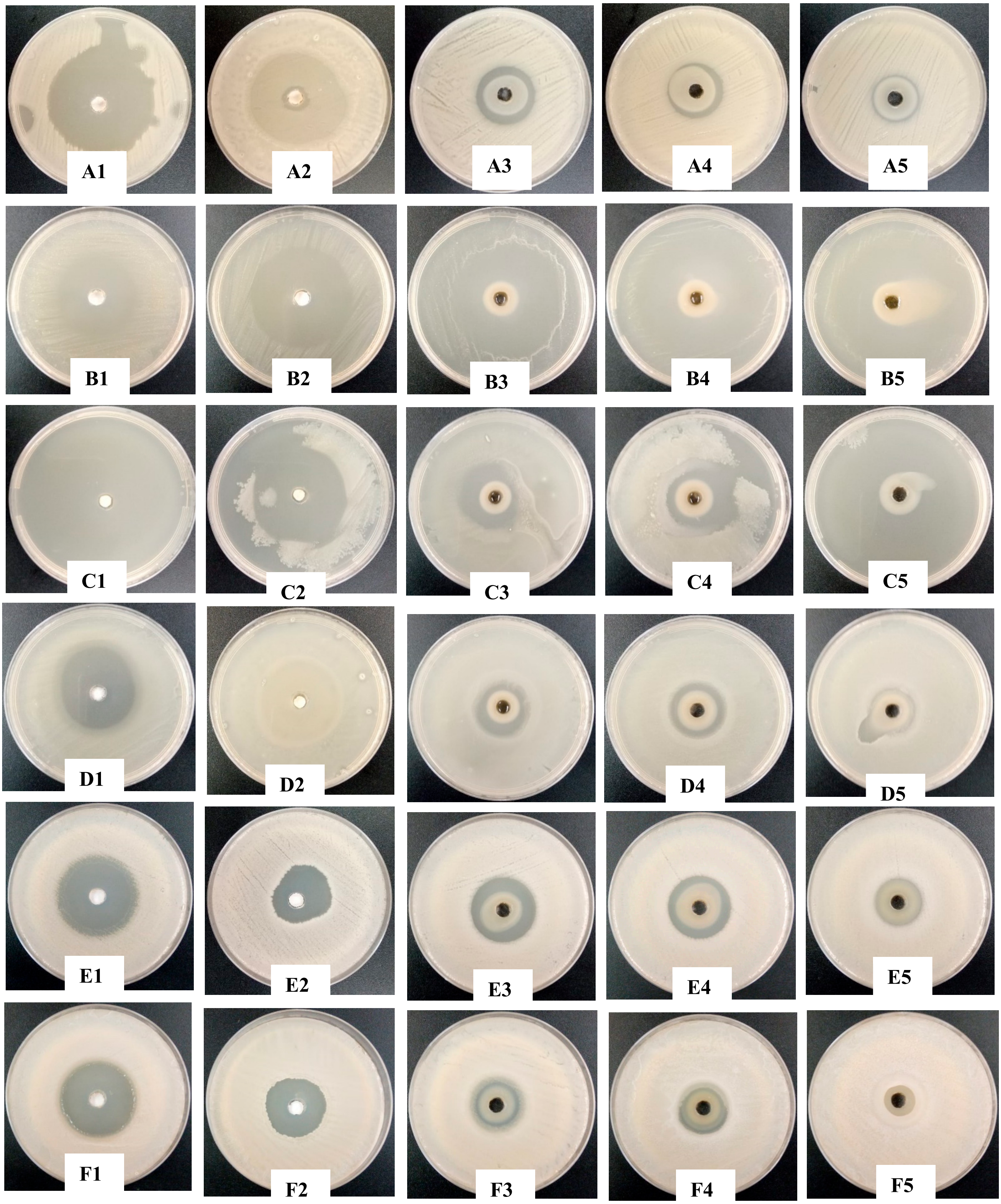

Figure S3: Inhibition of bacterial growth by cup plate experiment. A, B, C, D, E and F show inhibition of *Escherichia coli, Pseudomonas citronellolis*, *Pseudomonas aeruginosa,* Klebisella *sp, Bradyrhizobium japonicum and Ralstonia pickettii* respectively in the presence of 1- Ciprofloxacin, 2-Amoxacillin, 3-CHT$_{0.5}$-CuO nanocomposite, 4 – CHT$_{1.0}$-CuO nanocomposite and 5- CHT$_{2.0}$-CuO nanocomposite

Table S1: Assessment of the antimicrobial effect of CHT -CuO nanocomposites on gram positive and gram negative bacteria based on turbidity visualization of the culture and bacterial count on solid Agar plate. Values in brackets represent bacteria counts or colony forming units (CFU × 10^6/ml) after 24 h culture. Positive control presents culture liquid without bacterial inhibitor substance, and negative control is the culture without bacteria. '–' infers complete growth inhibition where '+' indicate bacteria growth.

Bacterium	$CHT_{0.5}$ -CuO nanocomposite Conc (%)					$CHT_{1.0}$ -CuO nanocomposite Conc (%)					$CHT_{2.0}$ -CuO nanocomposite Conc (%)					controls	
	10^{-1}	10^{-2}	10^{-3}	10^{-4}	10^{-5}	10^{-1}	10^{-2}	10^{-3}	10^{-4}	10^{-5}	10^{-1}	10^{-2}	10^{-3}	10^{-4}	10^{-5}	+ve	-ve
B. licheniformis	- (0)	++ (5.6×10^{5})	++ (9.5×10^{7})	+++ (1.1×10^{8})	++++ (1.4×10^{10})	- (0)	+ (5.6×10^{5})	++ (9.5×10^{7})	+++ (1.1×10^{8})	++++ (1.4×10^{10})	- (0)	++ (5.6×10^{5})	++ (9.5×10^{7})	+++ (1.1×10^{8})	++++ (1.4×10^{10})	++++ (1.8×10^{10})	- (0)
B. cerus	- (0)	- (0)	+ (6.4×10^{4})	+++ (1.2×10^{8})	++++ (1.3×10^{10})	- (0)	- (0)	+ (3.6×10^{3})	++ (8.0×10^{6})	+++ (1.6×10^{10})	- (0)	- (0)	+ (7.0×10^{3})	++ (3.0×10^{6})	++++ (1.9×10^{10})	++++ (2.5×10^{10})	- (0)
S. haemolitica	+ (3.0×10^{4})	++ (1.3×10^{7})	++ (1.8×10^{7})	++ (5.8×10^{7})	++++ (1.7×10^{12})	+ (6.8×10^{3})	++ (1.7×10^{7})	++ (1.1×10^{7})	++ (7.6×10^{7})	++++ (1.4×10^{12})	+ (7.0×10^{3})	++ (1.1×10^{5})	++ (7.6×10^{6})	++ (8.0×10^{7})	+++ (2.1×10^{8})	++++ (2.1×10^{12}	- (0)
M. luteus	- (0)	++ (3.2×10^{5})	++ (9.4×10^{7})	++ (5.8×10^{7})	+++ (1.7×10^{8})	- (0)	++ (5.6×10^{5})	++ (6.2×10^{5})	++ (3.4×10^{7})	+++ (1.8×10^{8})	- (0)	+ (7.2×10^{3})	++ (6.0×10^{4})	++ (4.7×10^{5})	+++ (2.1×10^{8})	++++ (8.6×10^{9})	- (0)
E. coli	- (0)	+ (3.6×10^{4})	++ (2.4×10^{7})	+++ (1.2×10^{8})	++++ (2.8×10^{10})	- (0)	+ (3.2×10^{4})	++ (8.0×10^{8})	+++ (7.1×10^{9})	++++ (1.9×10^{10})	- (0)	- (0)	++ (4.0×10^{4})	+++ (2.1×10^{8})	++++ (1.7×10^{10})	++++ (2.4×10^{10})	- (0)
P. citronellolis	- (0)	- (0)	++ (7.2×10^{4})	++ (8.0×10^{5})	++++ (2.3×10^{10})	- (0)	- (0)	++ (7.0×10^{4})	++ (3.6×10^{7})	++++ (1.5×10^{10})	- (0)	- (0)	++ (3.6×10^{4})	++ (1.9×10^{7})	++++ (1.9×10^{10})	++++ (1.9×10^{10})	- (0)
P. aeruginosa	- (0)	+ (2.0×10^{4})	++ (4.8×10^{6})	++ (8.8×10^{6})	++ (2.6×10^{7})	- (0)	+ (1.2×10^{4})	++ (5.8×10^{6})	+++ (2.8×10^{9})	+++ (8.0×10^{9})	- (0)	+ (4.4×10^{4})	++ (1.6×10^{7})	+++ (2.1×10^{8})	++++ (6.2×10^{9})	++++ (2.8×10^{10})	- (0)
Klebisella sp	- (0)	- (0)	++ (4.6×10^{4})	+ (9.8×10^{4})	++++ (2.4×10^{10})	- (0)	- (0)	+ (6.2×10^{3})	++ (1.4×10^{7})	++++ (1.9×10^{10})	- (0)	+ (2.8×10^{3})	++ (8.4×10^{6})	+++ (1.9×10^{8})	++++ (2.9×10^{10})	++++ (2.5×10^{10})	- (0)
B. japonicum	- (0)	- (0)	+ (3.4×10^{4})	++ (4.8×10^{7})	++++ (1.8×10^{10})	- (0)	- (0)	+ (6.4×10^{4})	++ (1.6×10^{7})	++++ (2.0×10^{10})	- (0)	+ (4.8×10^{4})	+ (7.6×10^{4})	+++ (1.9×10^{8})	++++ (2.1×10^{10})	++++ (2.3×10^{10})	- (0)
R. pickettii	- (0)	- (0)	+ (7.4×10^{4})	++ (5.4×10^{6})	++++ (1.9×10^{10})	- (0)	- (0)	+ (3.2×10^{4})	++ (1.6×10^{7})	++++ (2.5×10^{10})	- (0)	+ (4.0×10^{4})	++ (1.9×10^{7})	++ (1.6×10^{7})	++++ (2.6×10^{10})	++++ (2.9×10^{10})	- (0)

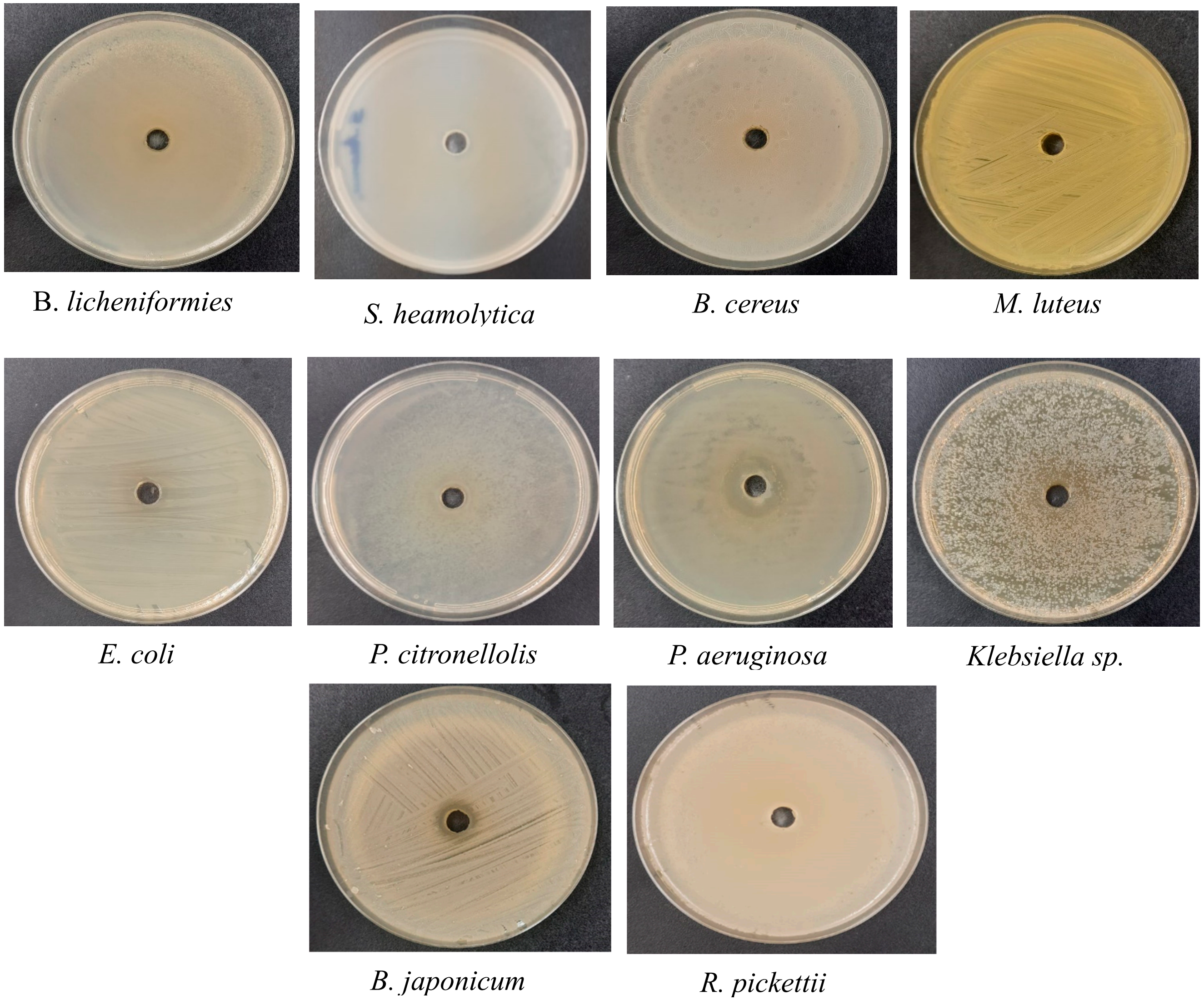

Figure S4: Inhibition of bacterial growth by cup plate experiment in the presence of OLE for all bacteria strains
